# The Good Food for Learning Universal Curriculum-Integrated Healthy School Lunch Intervention: Protocol for a Two-Year Matched Control Pre-Post and Case Study

**DOI:** 10.2196/30899

**Published:** 2021-09-21

**Authors:** Rachel Engler-Stringer, Jennifer Black, Nazeem Muhajarine, Wanda Martin, Jason Gilliland, Janet McVittie, Sara Kirk, Hannah Wittman, Amin Mousavi, Sinikka Elliott, Sylvana Tu, Brent Hills, Gordon Androsoff, Debbie Field, Brit Macdonald, Chelsea Belt, Hassan Vatanparast

**Affiliations:** 1 Department of Community Health and Epidemiology College of Medicine University of Saskatchewan Saskatoon, SK Canada; 2 Faculty of Land and Food Systems University of British Columbia Vancouver, BC Canada; 3 College of Nursing University of Saskatchewan Saskatoon, SK Canada; 4 Western University London, ON Canada; 5 Department of Educational Foundations College of Education University of Saskatchewan Saskatoon, SK Canada; 6 School of Health and Human Performance Dalhousie University Halifax, NS Canada; 7 Department of Educational Psychology and Special Education College of Education University of Saskatchewan Saskatoon, SK Canada; 8 Department of Sociology University of British Columbia Vancouver, BC Canada; 9 Saskatchewan Population Health Evaluation and Research Unit, University of Saskatchewan Saskatoon, SK Canada; 10 Saskatoon Public Schools Division Saskatoon, SK Canada; 11 CHEP Good Food Inc Saskatoon, SK Canada; 12 Coalition for Healthy School Food Food Secure Canada Montreal, QC Canada; 13 Little Green Thumbs Program Agriculture in the Classroom Saskatoon, SK Canada; 14 Health Promotion Department Saskatchewan Health Authority Saskatoon, SK Canada; 15 College of Pharmacy and Nutrition University of Saskatchewan Saskatoon, SK Canada

**Keywords:** school food programs, Canada, nutrition, intervention research, mHealth

## Abstract

**Background:**

Good nutrition affects children’s health, well-being, and learning, and schools offer an important setting to promote healthy behaviors that can last a lifetime. Once children reach school age, they spend more of their waking hours in school than in any other environment. Children’s eating habits may be easier to influence than those of adults. In Canada, households with children are more likely to experience food insecurity, and school food programs that are universally available to all children can support the development of healthy eating patterns across groups of varying socioeconomic status. There is a significant gap in the rigorous community-engaged academic research on the impact of school meal programs, especially universal ones.

**Objective:**

The aim of this population health intervention research is to study the impact of a 2-year universal, curriculum-integrated healthy school lunch program in elementary schools in Saskatoon, Saskatchewan, Canada, on food consumption, dietary quality and food and nutrition-related knowledge, attitudes, and practices.

**Methods:**

This population health intervention study will be conducted in 2 intervention elementary schools matched with 2 control schools. We will collect preintervention data, including objective measurements of food eaten at school and food-related knowledge, attitudes, and behaviors. This will be followed by the intervention itself, along with qualitative case studies of the intervention process in the 2 intervention schools. Then, we will collect postintervention data similar to the preintervention data. Finally, we will finish the data analysis and complete the ongoing sharing of learning from the project.

**Results:**

This study was funded in April 2020 but because of the COVID-19 pandemic, data collection did not begin until May 2021. The intervention will begin in September 2021 and end in June 2023, with end point data collection occurring in May and June 2023. The case study research will begin in September 2021 and will be ongoing for the duration of the intervention.

**Conclusions:**

The opportunity we have to systematically and comprehensively study a curriculum-integrated school lunch program, as well as the promising practices for school food programs across Canada, is without precedent.

**International Registered Report Identifier (IRRID):**

DERR1-10.2196/30899

## Introduction

### Background

Two events occurred in 2019 that made rigorous Canadian research on school food programs particularly needed and timely. First, the updated Canadian Food Guide (CFG) was released [1]. Its focus is on reducing the consumption of highly processed foods, encouraging consumption of a variety of healthy foods, shifting to plant proteins, eating as a social and conscious act, and cooking. Second, the March Federal Budget stated the following [2]:

Critically important for a child’s education is ensuring they have healthy meals before and during school. Currently, Canada has a mix of different school breakfast and lunch programs, but much more could be done. Budget 2019 announces the government’s intention to work with the provinces and territories towards the creation of a national school food program.

Ensuring that Canadians have the capabilities and opportunities to eat in accordance with the new CFG guidelines will be challenging, given that families struggle to consume healthy foods for multiple reasons including affordability, time scarcity, and food availability [3-7]. Debates have focused on how the new CFG recommendations can be achieved, with journalists and researchers stating that a national school food program might be a key approach [8].

Nutrition affects children’s health, well-being, and learning, and schools offer an important setting to promote health behaviors that can last a lifetime [9-13]. Households with children are more likely to experience food insecurity; however, recent research shows that the diet quality of Canadian children across the socioeconomic spectrum during school hours is poor [14]. School age children spend more of their waking hours in school than in any other environment, yet Canada is one of the only Organization for Economic Cooperation and Development countries without a national school food policy or program [15]. School food programs can include breakfast, lunch, and snacks, with or without integration into the curricula, and can contribute to child, family, and community health and well-being [16]. School food programs that are available to all children, regardless of a family’s ability to pay, can support the development of healthy eating patterns across groups of varying socioeconomic status (SES) [16,17].

The 160 members of the Canadian Coalition for Healthy School Food (CHSF), the largest Canadian organization advocating for school food programs, advocates for a universal school food program that is key to positively evaluated programs [17]. Universality means that all children have access to a school lunch program, and in the case of the intervention discussed here, at no cost, regardless of income. Most school breakfast and lunch programs in Canada are instead targeted to either self-identified or school-identified low-income families [18]. Discussions regarding a school food program for Canada are drawing largely on international evidence because of the limited Canadian data available [19,20]. There is a significant gap in rigorous academic research on the design of and outcomes of school meal programs, especially universal ones [21].

We conducted a scoping review of school food research in Canada to determine what we know from promising practices for school meal programs [20]. A total of 17 peer-reviewed and 19 gray literature articles discussed 24 programs in 10 provinces. There was one randomized control trial, no longitudinal studies, and one study with a control group. The breadth and depth of research on school food programs in Canada is lacking [19]. Upon analysis of the research to date, we concluded that the most promising programs for improved health address social determinants of health and teach about food systems and environmental sustainability.

The purpose of our population health intervention research (PHIR) is to study the process, benefits, and challenges of implementation, as well as impacts of a universal, curriculum-integrated, healthy school lunch program on diet quality and food and nutrition-related knowledge, attitudes, and practices (KAP) of elementary students. Universal school food programs reduce stigma compared with targeted programs, and Raine et al [22] found that only a minority of the intended target population is actually reached in targeted programs. Previous process evaluation studies have identified universal access and curriculum integration as key factors in the success of snack-based school food programs [23]. Curriculum-integrated means core curriculum (eg, math, science, and social studies) includes teaching aspects of food, nutrition, and food systems at the same time as providing the school lunch. Children also gain hands-on experience by growing some of the vegetables and preparing food for consumption.

This study is funded by the Canadian Institutes for Health Research and has undergone peer review (see Multimedia Appendices 1-4).

### Literature Review

#### Evolution of School Food Programs

The development of school food programs in high-income countries has followed three phases, the third of which is just taking hold today [16]. Globally, school food programs were often established primarily to reduce hunger [16]. In the 1970s, in some parts of Europe and in the 1990s and the 2000s in the United States of America and the United Kingdom, a shift toward improving food quality began, creating the second phase of school food programs. This second phase shifted the focus toward dietary guidelines for school food programs to improve the nutritional quality of food served. The third phase, in its infancy in most countries (Canada included), is a response to increased rates of childhood obesity and nutrition-related chronic diseases, a larger societal context of challenges in the food system, climate change, and environmental degradation. This phase incorporates food systems and societal issues into food programs and policies and generally integrates them more closely with curricula and the school environment as a whole [16,24]. Various researchers argue that this approach is the future of school food programs around the world and is consistent with the principles outlined in the CFG [25,26]. We recently examined international literature on school food programs in high-income countries and outlined key characteristics needed in a national school food program for Canada, including universality, curriculum integration, adaptation to local contexts, and sustainable funding [21].

#### School Food Programs, Nutrition, and Health

International research on the health and dietary behavior impacts of school food programs in high-income countries has found modest positive effects overall, including higher vitamin intake and increased vegetable and fruit consumption, especially in younger children [27-30]. Studies from various countries that compare the nutritional quality of food consumed at school that was brought from home versus food acquired through school food programs has found that school food programs provide healthier food overall (regardless of the SES of child participants) [31-38].

Only a small proportion of Canadian children meet the Canadian eating recommendations, with low vegetable and fruit consumption of particular concern [39,40]. Research on overall dietary patterns in Canada highlights the very large proportions of ultraprocessed foods high in salt, sugar, and some fats being consumed [41,42]. Families struggle to introduce minimally processed healthy foods for a variety of reasons [3-5].

Provision of healthy school lunches is challenging in the context of parents working long hours [43,44], and families are struggling to adopt healthy food behaviors [44]. Parents may rely on highly processed foods, low in key nutrients but high in salt, sugar, and fat, to cope with demands on time [45,46]. The diets of Canadian children across the socioeconomic spectrum are poor [47]. There is some evidence from the United States that school food programs can reduce disparities in vegetable and fruit consumption between children from higher versus lower SES households and limit the intake of minimally nutritious foods among higher SES households [48]. Introduction of healthy foods in a universal school food program could provide all children with greater opportunities to learn about and eat healthy foods in a way that is not stigmatizing [49].

Our team’s previous observational research on school food in and around Saskatoon, Saskatchewan, Canada, characterized lunches using a school food checklist and digital photography in randomly selected urban schools with a meal program (n=3), urban schools without a meal program (n=3), and rural schools without a meal program (n=3) [50]. The number of servings of each CFG food group was determined and compared with one-third of the recommended daily intake. The Healthy Eating Index (HEI) scores were calculated. In results similar to Tugault-Lafleur et al [14] using the national Canadian Community Health Survey data for the school day, just over half of the students who brought lunches from home met the recommendations for grain products and meat and alternatives, less than one-third met recommendations for vegetables and fruits, under one-fourth met the recommendations for whole grains, and even fewer met the recommendations for milk and alternatives. The HEI scores of students in meal programs were greater than those of students who brought lunches from home because they included more whole grains, met meat and alternatives recommendations, and contained fewer calories from minimally nutritious foods (high-fat and high-sugar foods such as candies, chocolate, and sauces). Children not participating in meal programs brought about one-third of calories as minimally nutritious food, about double that of meal program student lunches.

There are numerous reports of school staff and other stakeholders expressing a desire to offer universal lunch programs rather than the more common targeted programs and expressing concern about the quality of food currently consumed by Canadian children at school outside of lunch programs [51,52]. Drawing on international evidence, an evaluation was conducted of a 2-year pilot of free school meals in 3 local authorities in the United Kingdom [53]. Two local authorities made free school meals universal to all primary school children, whereas the third extended free school meal entitlements to a larger number of students but was not universally free. In the extended entitlement (not universal) authority, there were no impacts found on children’s eating habits, whereas in the universal entitlement authorities, there were reductions in the consumption of potato chips and soft drinks and an increase in vegetables consumed. In the universal pilot areas, parents’ perceptions of school meals for health were more positive and they thought that their children were more willing to try new foods.

#### Learning Outcomes and School Food Programs

Educational achievement is associated with health across life span [54]. Studies on school food programs and academic achievement, attendance, tardiness, and dropout rates point to important impacts of school food programs. Attendance and tardiness appear to be the educational outcomes with the most available evidence, but some studies have found improvements in academic achievement with the introduction of school food programs [55-65]. School food programs can also contribute to teaching culinary heritage, social norms around food, and environmental sustainability [26,66,67]. We are conducting a separate study in partnership with educational research colleagues on educational outcome changes as a result of this intervention.

#### Food Literacy, Environmental Education, and School Food Programs

Various countries, including Canada most recently, have adopted nutritional recommendations that recognize the connections between nutrition and environmental sustainability [1,68]. According to Cullen et al [69], food literacy is “the ability to make decisions to support the achievement of personal health and a sustainable food system considering environmental, social, economic, cultural, and political components.” School food programs focused on the provision of healthy, sustainable foods (including vegetables and fruit, plant proteins, and locally produced foods) along with the promotion of sustainable food behaviors such as school gardening and learning how to reduce food waste, may work together to change food behaviors [26], which may spill into life away from school [70]. Stone [71] and Weaver-Hightower [72] explain how children can be involved in age-appropriate ways to grow and prepare food, along with learning how the food system works and its environmental and social challenges, to integrate learning with a meal program. This integration enables appreciation for food and a greater willingness to try new foods [73,74].

The evidence for school food interventions that include both meals and curriculum is getting stronger internationally, but not much Canadian evidence is available [19,20,62,75]. These interventions include introducing healthy foods, integrated with the curriculum, and with parent involvement [28,76]. They take an education-integrated approach that involves children in growing and preparing food, teaching about food system sustainability, and healthy behaviors [26]. Internationally, Oostindjer et al [26] juxtapose the Swedish and British school food programs and the Japanese, French, Italian, and Finnish school food programs on this front. The Finnish universal lunch program, which is a key aspect of their education system, is integrated with the curriculum, including learning objectives related to social relationships and eating norms that are lacking in Sweden and the United Kingdom [77-79]. According to a review of international literature, this emerging integration of school meals with classroom curricula aligned with food cultural learning and establishing an optimal food and social environment may facilitate learning of healthy and sustainable food behaviors [26].

## Methods

### Overview

This multimethod PHIR is informed by the theory of change, where the ways in which a program brings about specific outcomes are explicitly described, as is commonly done with complex population health program evaluations [80]. We have also developed a program logic model for population health intervention as part of our theory of change. The overall research design is a case study with an embedded matched control pre and post study of a universal, curriculum-integrated school lunch program in elementary schools consistent with promising practices in school food programs for Canada [19,26,75]. The case study will fill an important research gap in rigorous PHIR that is critical to inform policy change, given that it examines both implementation and health-related outcomes [81-83]. Our design includes the following:

1. A comprehensive case study of the process of implementation, benefits, challenges, and perceptions of a universal, curriculum-integrated school lunch program including key informant and caregiver interviews and structured and unstructured observations of the intervention school food environment.
2. An embedded, nonblinded, experimental study with a control group including an assessment of plate-waste of lunches to examine contribution to overall diet quality (based on a third of daily food consumption [14]) and surveys of food-related KAP.

### Research Objective and Research Questions

The objective of our study is to examine the process of implementation and the impacts of a universal, curriculum-integrated school lunch program in 2 schools by responding to the following research questions (RQs):

- What are the benefits of and challenges faced in establishing and administering a universal curriculum-integrated healthy school lunch program?
- What is the difference in diet quality, food group, and key nutrient consumption among children in intervention schools after the implementation of a comprehensive, curriculum-integrated universal school lunch program as compared with children in control schools?
- What is the difference in food-related KAP among child participants in intervention schools after the implementation of a comprehensive, curriculum-integrated universal school lunch program as compared with children in control schools?
- What aspects of curriculum integration show promise in enhancing food-related learning?

### Hypotheses

Owing to the qualitative nature of the case study RQs, we do not have hypotheses for them. For the embedded experimental study, we hypothesize that there will be significant differences in food consumption and dietary quality between intervention and control schools at the end point, with children in intervention schools having higher consumption of vegetables and fruits, whole grains, and dairy products and lower consumption of minimally nutritious foods. We expect differences in food-related KAP of children in intervention versus control schools, with intervention school participants having greater knowledge and more positive attitudes and practices with regard to nutritious and sustainable food.

### Sample

We conducted a sample size calculation that indicated that we needed a minimum sample of 148 children from 2 intervention schools matched with 148 children from 2 control schools to detect changes in eating behavior, accounting for attrition from the baseline (pretest) to the end point (posttest). Furthermore, 2 intervention and 2 control elementary schools were selected by the Saskatoon Public Schools Division (SPSD) from the 8 schools that currently have small lunch programs and the infrastructure to scale up to serve the whole school. Saskatoon is a midsized Canadian Prairie city with a population of approximately 325,000 residents. The 4 schools were selected from among the 8 schools because the school staff in these schools are willing to participate in this initial intervention study which is critical at this stage. The schools are located in lower SES neighborhoods with more ethnic diversity including relatively large numbers of newcomers and Indigenous children compared with other neighborhoods in Saskatoon [84].

Our sample of students in the intervention schools is a 1:1 matched sample of children from 2 control schools based on self-identified gender, age, school grade, and school neighborhood median income, who will be followed from the baseline to the end point (2 full school years with a summer break in between). All children in grades 1-6 in each school will be invited to participate (approximately 170 students in each school in these grades). Our total sample from which participants will be drawn is about 340 (170 in each school), much more than the minimum 148 needed in our sample size calculation. They will be matched with an equivalent number in control schools.

### Intervention Details

The intervention comprises (1) the universal school lunch program and (2) the associated curriculum. The intervention will last 2 full school years (plus a summer between) to allow time for the schools to adapt to new food and curricular practices. Case study research will be ongoing over 20 months of school.

The school lunch will be offered every day at no cost to all students in the intervention schools and will focus on nutritious foods, including a variety of vegetables and fruits, whole grains, dairy products, plant proteins, and some meat. With SPSD staff and CHSF input, we developed a 6-week menu for the school food program with considerations for cultural appropriateness and the new CFG recommendations, including more plant-based proteins and whole grains. We are working with a community-based organization that works with local producers to incorporate local foods where possible into school food menus. The menu includes a variety of vegetables and fruits because they are particularly low in the diets of Canadian children [14,46], and it has been reviewed by a dietitian.

For curriculum integration, we adapted 6 lesson plans each from kindergarten up to grade 6 (ages 5-12 years) to teach food safety, food preparation, nutrition, gardening, and food waste reduction tied to the Saskatchewan curriculum and informed by our scoping review. The curriculum was developed with the CHSF and several Saskatchewan teachers and is ready to use in the classroom with little additional preparation by teachers. In addition to the lesson plans, all classrooms in each grade will also have two experiences involved in cooking: one indoor and one outdoor food-growing experience. Consistent with best education practices, our focus is on a hands-on curriculum.

### Data Collection and Analysis

#### Phase 1: Intervention Planning

We have ethical approval in place (University of Saskatchewan BEH-509) and a memorandum of understanding prepared between principal investigator RES and the SPSD. In 2018, we collected baseline data on school food environments in the 2 intervention schools to inform study preparation. Intervention planning was supposed to be conducted from March to May 2020; however, because of the COVID-19 pandemic, it was put off and conducted between January and May 2021. The COVID-19 pandemic-related prohibitions on field data collection also affected this study, and permission to enter schools was granted in mid-May 2021.

#### Phase 2 and Phase 4: Baseline and End Point Measurement

RQ2: What is the difference in diet quality, food group, and key nutrient consumption among children in intervention schools after the implementation of a comprehensive, curriculum-integrated universal school lunch program as compared with children in control schools?

A digital photography–enhanced plate-waste study will assess food eaten for lunch by students in intervention and control schools (n=148 minimum each for a total of 296; 4 schools) on 2 days at baseline (preintervention) and then on 2 days at the end point (in the last 2 months of the intervention). Each participating child will have four measurements of plate-waste data (two baseline and two end point data). We will follow the protocol developed by coinvestigator HV, which has been extensively used in studies of school-aged children [85-87]. The procedures for data collection involve weighing the served food and leftovers, using digital photography and a food scale during the lunchtime with an app installed on tablets. This method is considered the most precise measurement of dietary intake compared with other dietary assessment methods that are typically used, such as food records [88,89]. The literature indicates no significant impact of the method on eating behaviors in children [88,89]. Digital photography of plate-waste will be conducted by 2 research assistants in half the school classrooms on each data collection day, for a total of 4 data collection days at both baseline and at end point in each school. The practice of collecting data from half the classrooms in a single school on each data collection day will ensure minimal disruption of regular school days.

Our plate-waste approach allows us to infer the amount of food consumed by calculating the difference between the amount served and the amount left over [88,90]. At the end point, the information about the menus (recipe level data) on the days of data collection will be entered in the app on tablets before lunch. A label with the identification number of the child is placed on the tableware to correctly identify each child’s meal and leftovers and scanned at the time of data collection for each child. A photograph (62 cm away from the food, at a 45° angle [91]) is taken of student’s tableware (ie, each container in a lunch box, plates, or other) to visually capture the size and general appearance of items to subtract them from the food consumed. Each entry contains the child’s identification number, the photo forms to record the weight of the food, and whether the photo was taken before or after the child’s meal. Leftover food is weighed and photographed again at the end of the meal. By subtracting the weighed leftovers from the weighed food served, the food consumed will be obtained. The information pertaining to energy, macronutrients, and micronutrients consumed will be derived using Food Processor nutrition analysis software (The Food Processor, Esha Research version 11.3.285). The final report will be exported to Microsoft Excel where food groups based on the new CFG will be calculated (new recommendations on the amount and type of food taken by age group are not available as of June 2021 but will be used once released by Health Canada).

The collated baseline and end point data will go through data cleaning, processing, and quality control using protocols developed in the Vatanparast Nutritional Epidemiology laboratory. After creating the master data file including original and derived variables of interest, such as the Nutrient Rich Food index (9.3) [92], HEI [14] based on the new CFG, sex assigned at birth, self-identified gender, age, grade, and neighborhood of residence as a proxy for SES, the descriptive analyses will provide the initial information on these variables. Categorical variables (ie, HEI categories) will be summarized in frequency tables. Regression models will examine changes from baseline to the end point across key outcome variables including the Nutrient Rich Food index scores, HEI scores, CFG servings, and key nutrients (calcium, sodium, vitamin D, folate, and calories from minimally nutritious food) and whether changes differ between the control and intervention schools. Final models will adjust for potentially confounding factors including sex assigned at birth, self-identified gender, age, grade, neighborhood of residence, and baseline dietary intake (for more detail on identifying a priori potential confounders and controlling for them, see *Phase 5: Final Analysis and Dissemination* section below). We will also examine potential interaction effects between the intervention and control groups and age, sex, and gender (as detailed above in the sample description) to assess whether the effect of the intervention on dietary outcomes was modified by key student-level characteristics. Clustering of students within the classroom and schools (nonindependence) will be taken into account by applying random-effects multilevel modeling approaches to data.

RQ3: What is the difference in food-related KAP among child participants in intervention schools after the implementation of a comprehensive, curriculum-integrated universal school lunch program as compared with children in control schools?

At baseline and at the end point in both intervention and control schools, we will administer a survey of food-related KAP adapted from the Individual Eating Assessment Tool (I-EAT) to all participating students in grades 4-6 in the intervention and control schools. According to the KAP model, knowledge (K) accumulates and attitudes (A) change and these changes promote changes in practices (P) over time. We cannot administer the survey to children younger than grade 4 because of inconsistent literacy levels before that age and older than grade 6 because they will no longer be at the school at the end point. Given our expected 80% response rate, we will have a sufficiently large sample in these three grades only to carry out these analyses. This survey was originally developed by Black et al [47,93-95] and we have adapted and piloted it with 100 children. We conducted a factor analysis of the pilot results for validation and then made some additional minor changes following the analysis, specifically removing four questions that did not add relevant data. We included I-EAT questions on food, cooking and nutrition knowledge and beliefs, confidence in participating in various food-related activities, attitudes toward whole grains, vegetable and fruit consumption, participation in learning about food, nutrition and cooking, confidence in engaging in food-related activities, engagement in food-related sustainability practices, and demographics.[47,93,94] We will analyze I-EAT derived outcome data and compare the differences at end point between the intervention and control student data.

#### Further Quantitative Analyses

Analyses of plate-waste data and survey data will be conducted using the intention-to-treat principle [96]. To assess the effect of the intervention, we will use mixed-effect models using time of measurement (baseline or end point), group (intervention or control), and an interaction between time and group as fixed effects (base model). The neighborhood of residence (proxy for SES) will be used to determine whether SES influences the response to the intervention. To account for clustering related to repeated measures and because of sampling of students within classes and schools, variables representing participants and schools will be included as random effects in all models. Additional models will account for potentially confounding variables identified using directed acyclic graphs for each outcome [97,98]. Directed acyclic graphs are used to identify potential confounders using prespecified conceptual diagrams and testing these using the data to increase the precision of estimates and causal paths based on regression models. Analyses will be conducted using the MIXED procedure in SAS version 9.4 (SAS Institute, Inc).

#### Phase 3: Qualitative Data Collection and Analysis

RQ1: What are the benefits and challenges faced in establishing and administering a universal, curriculum-integrated healthy school lunch program?

RQ4: What aspects of curriculum integration show promise in enhancing food-related learning?

We will collect qualitative data from the 2 intervention schools. In the case study research, the investigator explores a bounded system or case (a school) over time through detailed data collection, drawing on multiple sources of information [99,100]. We have already conducted preintervention research in the intervention schools examining the school food environment using a tool called the School Food Environment Assessment Tool (SF-EAT) [101]. SF-EAT assesses the integration of healthy and sustainable food strategies in the school food environment, including food availability, food preparation facilities, gardening, composting, integration of food issues into the curriculum, and availability of healthy and environmentally sustainable food [101]. The administration of the SF-EAT tool was complemented with key informant interviews on barriers and facilitators for adopting the curriculum-integrated lunch program.

We will use semiparticipant observation and key informant interviews with school staff to evaluate the process of implementing the curriculum-integrated school lunch programs in the 2 intervention schools. We will study 3-5 individual classrooms in different grades to detail the aspects of each case. Observation will occur during (1) meetings when school staff are planning for the establishment of the school lunch program; (2) meetings when school staff are discussing curricular integration; (3) during lunch preparation, service, and clean-up; (4) when children are involved in preparation; and (5) during other curriculum integration activities such as during lessons involving growing vegetables. Observations will occur at minimum for 1 school day each month in each school and at school staff meetings as needed during the intervention period, with key informant interviews occurring throughout. Observations will be recorded using voice memos and field notebooks, which will be transcribed or detailed upon leaving the school at the end of the day. We will focus our data collection on how the intervention is being carried out, including challenges experienced and its fidelity to the original intervention design. We will interview caregivers on their perceptions of the program and behavioral changes they identify, including caregivers of children who have never participated in a school food program and those who have, across grades.

The qualitative data will document how schools establish a universal lunch and parent perceptions of the program. Our observations and interview questions will focus on the perceived benefits and challenges of establishing and administering a universal, curriculum-integrated healthy school lunch program and on the aspects of curriculum integration that appear to show promise in terms of enhancing food-related learning. The cases will inform the establishment of programs on a larger scale and in other contexts.

Interviews will be audio-recorded, transcribed verbatim, and analyzed using NVivo software (QSR International) for coding and theme development. Semiparticipant observation data will be recorded as field notes during observation as well as immediately after. Qualitative data will be analyzed initially in an open coding process to allow for emerging themes, followed by analysis using a priori generated code lists derived from the literature and our RQs. Our analytical approach will build concepts and themes inductively, testing and refining them with participants [102]. This hybrid inductive and deductive thematic analysis has been described by Fereday and Muir-Cochrane [103]. A regular debriefing with the research team and key school informants will assist in the refinement of concepts and themes. These steps will strengthen the credibility and dependability of the results [104,105]. Our contextualized interpretation and meaning making with stakeholders is consistent with Moss and Dahlberg [106] for research in educational contexts.

#### Phase 5: Final Analysis and Dissemination

We will enter, clean, and analyze postintervention data. This phase will include end-of-study knowledge translation (KT), although because of the existing collaborative relationship between the academic and nonacademic partners, KT will be ongoing throughout the entire study. Although this study is about filling gaps in the academic literature, it is also about ensuring practice relevance. Everything from conceiving the study to the development of RQs and practices has been and will be conducted closely with the school division, the CHSF, and other partners.

We have experience with integrated community-university KT strategies, which have generated practice and policy change [107-110], and we will apply what we have learned [111,112]. The inclusion of knowledge users or decision makers as members of the research team will facilitate evidence-informed decision-making and integrated knowledge transfer and exchange. This collaborative approach informs and directs our research and sharing of results and other outputs and is consistent with the Canadian Institutes of Health Research Guidelines for Research with Aboriginal People [113]. Although the focus of this research is not Indigenous people, we believe that these guidelines are relevant for all community-based research. We will work closely with our partners to conduct KT as needed and requested. Materials will be developed and designed with the goal of their use in future programming.

## Results

This study was funded in April 2020, but because of the COVID-19 pandemic, data collection did not begin until May 2021. The intervention will begin in September 2021 and end in June 2023, with end point data collection occurring in May and June 2023. The case study research will begin in September 2021 and will be ongoing for the duration of the intervention.

## Discussion

### Principal Findings

In real-world settings, there are a number of potential challenges to this research, some we can anticipate now and some we will have to address as they come up. Fortunately, our team has a wealth of experience conducting research with children and school staff in the school environment. First, the research team is also an intervention team. As we are piloting a type of universal, curriculum-integrated intervention that is quite innovative, we do not think that operating separately would be either feasible or appropriate. Part of the strength of this research is the collaboration, and we will draw on this with the intention that future research, once the intervention has benefited from this intensive study, will be conducted separately from the intervention.

We have already encountered the challenge of the COVID-19 pandemic. This project was funded in April 2020; however, because of school closures between March and June 2020, and the uncertainty of opening schools during a pandemic, the project was delayed by a year. Baseline data were collected in May and June of 2021. The intervention will begin in September 2021 with the new school year, but as of July 2021, we do not know what new challenges will exist with case study data collection because of the pandemic. We will need to adapt our protocols to whatever public health measures are in place over the 2 intervention school years.

We may encounter challenges with curriculum integration with teachers and other school staff. To minimize this, we have developed an intervention and research with the school division and have already been collecting baseline data in the intervention schools on current practices and needs. We have practiced limited family and teacher engagement (due to the COVID-19 pandemic) within the schools and will adapt as needed to ensure that both families and teachers continue to participate.

Next, although plate-waste studies are considered to be the best approach available for collecting nutrition data, there are limitations to conducting them in school settings. At baseline, weighing and photographing the foods brought from home before anything has been eaten at lunchtime and then again after is relatively straightforward in our considerable experience, but that would not capture any food shared between child participants nor food eaten before and after. It is also difficult to capture anything eaten outside of the lunch program as plate-waste data collection must occur before and after eating lunch only. This may mean that we slightly underrepresent what is eaten at school. We will test our method by comparing observations conducted during the case study with the plate-waste data at both the baseline and at the end point.

There are limitations to what we can infer from the measurement of KAP, but this is the most appropriate approach for a study at this stage. We are not measuring aspects of health status beyond nutritional outcomes. We are also collecting only interview data on the perceptions of the program from parents. We have limited our study in this way to keep it feasible but are working on efforts to examine additional aspects of health.

### Conclusions

Numerous national organizations are calling for a Canadian school food program [52,114]. Up until now, hundreds of ad hoc programs have been operating across the country. Although they contribute to the health of participating children, a national program has the potential to support the health and learning of all Canadian children. The opportunity we have is to systematically and comprehensively study a curriculum-integrated school lunch program without precedent. We have assembled a very strong team and a supportive research environment to address complex issues, apply innovative approaches, and translate our research findings into action.

## Appendix

Multimedia Appendix 1 Scientific officer notes.

Multimedia Appendix 2 Notice of decision.

Multimedia Appendix 3 Ranking and funding decision.

Multimedia Appendix 4 Complete reviews.

## Abbreviations

CFG: Canadian Food Guide
CHSF: Coalition for Healthy School Food
HEI: Healthy Eating Index
I-EAT: Individual Eating Assessment Tool
KAP: knowledge, attitudes, and practices
KT: knowledge translation
PHIR: population health intervention research
RQ: research question
SES: socioeconomic status
SF-EAT: School Food Environment Assessment Tool
SPSD: Saskatoon Public Schools Division
