# Correction: The Good Food for Learning Universal Curriculum-Integrated Healthy School Lunch Intervention: Protocol for a Two-Year Matched Control Pre-Post and Case Study

**DOI:** 10.2196/34393

**Published:** 2021-11-03

**Authors:** Rachel Engler-Stringer, Jennifer Black, Nazeem Muhajarine, Wanda Martin, Jason Gilliland, Janet McVittie, Sara Kirk, Hannah Wittman, Amin Mousavi, Sinikka Elliott, Sylvana Tu, Brent Hills, Gordon Androsoff, Debbie Field, Brit Macdonald, Chelsea Belt, Hassan Vatanparast

**Affiliations:** 1 Department of Community Health and Epidemiology College of Medicine University of Saskatchewan Saskatoon, SK Canada; 2 Faculty of Land and Food Systems, University of British Columbia Vancouver, BC Canada; 3 College of Nursing, University of Saskatchewan Saskatoon, SK Canada; 4 Western University London, ON Canada; 5 Department of Educational Foundations, College of Education, University of Saskatchewan Saskatoon, SK Canada; 6 School of Health and Human Performance, Dalhousie University Halifax, NS Canada; 7 Department of Educational Psychology and Special Education, College of Education, University of Saskatchewan Saskatoon, SK Canada; 8 Department of Sociology, University of British Columbia Vancouver, BC Canada; 9 Saskatchewan Population Health Evaluation and Research Unit, University of Saskatchewan Saskatoon, SK Canada; 10 Saskatoon Public Schools Division Saskatoon, SK Canada; 11 CHEP Good Food Inc Saskatoon, SK Canada; 12 Coalition for Healthy School Food, Food Secure Canada Montreal, QC Canada; 13 Little Green Thumbs Program, Agriculture in the Classroom Saskatoon, SK Canada; 14 Health Promotion Department, Saskatchewan Health Authority Saskatoon, SK Canada; 15 College of Pharmacy and Nutrition, University of Saskatchewan Saskatoon, SK Canada

In “The Good Food for Learning Universal Curriculum-Integrated Healthy School Lunch Intervention: Protocol for a Two-Year Matched Control Pre-Post and Case Study” (JMIR Res Protoc 2021;10(9):e30899), one error was noted.

In the originally published paper, one author, Sylvana Tu, was not included in the list of authors. Sylvana Tu has now been included in the authorship list between authors Sinikka Elliott and Brent Hills. Sylvana Tu's author affiliation has also been added as follows:

Saskatchewan Population Health Evaluation and Research Unit, University of Saskatchewan, Saskatoon, SK, Canada

This affiliation has been added as affiliation 9 in the corrected paper, and the remaining affiliations have been renumbered accordingly.

The full list of authorship and affiliations was as follows in the originally published paper:

Rachel Engler-Stringer^1^, PhD; Jennifer Black^2^, PhD; Nazeem Muhajarine^1^, PhD; Wanda Martin^3^, PhD; Jason Gilliland^4^, PhD; Janet McVittie^5^, PhD; Sara Kirk^6^, PhD; Hannah Wittman^2^, PhD; Amin Mousavi^7^, PhD; Sinikka Elliott^8^, PhD; Brent Hills^9^, MEd; Gordon Androsoff^10^, MSc; Debbie Field^11^, MA; Brit Macdonald^12^, BA; Chelsea Belt^13^, MSc; Hassan Vatanparast^14^, PhD

^1^Department of Community Health and Epidemiology, College of Medicine, University of Saskatchewan, Saskatoon, SK, Canada

^2^Faculty of Land and Food Systems, University of British Columbia, Vancouver, BC, Canada

^3^College of Nursing, University of Saskatchewan, Saskatoon, SK, Canada

^4^Western University, London, ON, Canada

^5^Department of Educational Foundations, College of Education, University of Saskatchewan, Saskatoon, SK, Canada

^6^School of Health and Human Performance, Dalhousie University, Halifax, NS, Canada

^7^Department of Educational Psychology and Special Education, College of Education, University of Saskatchewan, Saskatoon, SK, Canada

^8^Department of Sociology, University of British Columbia, Vancouver, BC, Canada

^9^Saskatoon Public Schools Division, Saskatoon, SK, Canada

^10^CHEP Good Food Inc, Saskatoon, SK, Canada

^11^Coalition for Healthy School Food, Food Secure Canada, Montreal, QC, Canada

^12^Little Green Thumbs Program, Agriculture in the Classroom, Saskatoon, SK, Canada

^13^Health Promotion Department, Saskatchewan Health Authority, Saskatoon, SK, Canada

^14^College of Pharmacy and Nutrition, University of Saskatchewan, Saskatoon, SK, Canada

This has been corrected to:

Rachel Engler-Stringer^1^, PhD; Jennifer Black^2^, PhD; Nazeem Muhajarine^1^, PhD; Wanda Martin^3^, PhD; Jason Gilliland^4^,PhD; Janet McVittie^5^, PhD; Sara Kirk^6^, PhD; Hannah Wittman^2^, PhD; Amin Mousavi^7^, PhD; Sinikka Elliott^8^, PhD; Sylvana Tu^9^, MPH; Brent Hills^10^, MEd; Gordon Androsoff^11^, MSc; Debbie Field^12^, MA; Brit Macdonald^13^, BA; Chelsea Belt^14^, MSc; Hassan Vatanparast^15^, PhD

^1^Department of Community Health and Epidemiology, College of Medicine, University of Saskatchewan, Saskatoon, SK, Canada

^2^Faculty of Land and Food Systems, University of British Columbia, Vancouver, BC, Canada

^3^College of Nursing, University of Saskatchewan, Saskatoon, SK, Canada

^4^Western University, London, ON, Canada

^5^Department of Educational Foundations, College of Education, University of Saskatchewan, Saskatoon, SK, Canada

^6^School of Health and Human Performance, Dalhousie University, Halifax, NS, Canada

^7^Department of Educational Psychology and Special Education, College of Education, University of Saskatchewan, Saskatoon, SK, Canada

^8^Department of Sociology, University of British Columbia, Vancouver, BC, Canada

^9^Saskatchewan Population Health Evaluation and Research Unit, University of Saskatchewan, Saskatoon, SK, Canada

^10^Saskatoon Public Schools Division, Saskatoon, SK, Canada

^11^CHEP Good Food Inc, Saskatoon, SK, Canada

^12^Coalition for Healthy School Food, Food Secure Canada, Montreal, QC, Canada

^13^Little Green Thumbs Program, Agriculture in the Classroom, Saskatoon, SK, Canada

^14^Health Promotion Department, Saskatchewan Health Authority, Saskatoon, SK, Canada

^15^College of Pharmacy and Nutrition, University of Saskatchewan, Saskatoon, SK, Canada

The correction will appear in the online version of the paper on the JMIR Publications website on November 3, 2021, together with the publication of this correction notice. Because this was made after submission to PubMed, PubMed Central, and other full-text repositories, the corrected article has also been resubmitted to those repositories.

