# Supplementary material for: The Good Food for Learning Universal Curriculum-Integrated Healthy School Lunch Intervention: Protocol for a Two-Year Matched Control Pre-Post and Case Study
Source: JMIR Res Protoc. 2021 Sep 21;10(9):e30899. doi: 10.2196/30899 (PMC8493466; doi:10.2196/30899)
Supplement: Multimedia Appendix 1 [file resprot_v10i9e30899_app1.pdf]

January/Janvier 22, 2020

DC0190GP

Canadian Institutes of Health Research / Instituts de recherche en santé du Canada

Notice of Decision / Avis de décision

Application Number/Numéro de la demande: 425692

Committee Code/Code du comité: PH1

Applicants/Candidats: Dr. Rachel Rosa Engler-Stringer Ms. Debbie Ethel Field

With/Avec: Dr. J. Black, Dr. J. Gilliland, Dr. S. Kirk, Dr. W. Martin, Dr. A. Mousavi, Dr. N. Muhajarine, Dr. H. Vatanparast, Dr. H. Wittman

Institution paid/ Établissement payé: University of Saskatchewan

Title/Titre: Improving School Food: Universal Integrated Healthy Lunch Intervention Research

Primary Inst./ Inst. principal: Population and Public Health / Santé publique et des populations

Other Related Inst./ Autres inst. connexes: Nutrition, Metabolism and Diabetes / Nutrition, métabolisme et diabète

**Competition Outcome/Résultats du concours:**	Project Grant / Subvention Projet<br>September/Septembre 11, 2019
**Number in competition/Nbre de demandes dans le concours:**	2183
**Number approved/Nbre de demandes approuvées:**	385

**Decision on your application/ Décision sur votre demande:**	Approved / Approuvée
**Total Funding Amount:/ Montant total du financement:**	$527,849
**Term/Durée:**	**3 yrs/ans 6 months/mois**

Peer Review Committee Recommendation, for your information and use/
Recommandation du comité d'examen par les pairs, pour fins d'information et d'utilisation:

**Committee/Comité:**	Public, Community & Population Health / Santé publique, santé communautaire et santé des populations
**Number reviewed/ Nbre de demandes examinées:**	47
**Number approved in that committee/ Nbre de demandes approuvées dans ce comité:**	9
**Application rank within the committee/ Rang de la demande dans ce comité:**	5
**Percent Rank Within the Committee/ Rang en pourcentage au sein du comité:**	91.3%
**Rating/ Cote:**	4.48

This document is for information only. Official payment is stated on the CIHR Authorization for Funding.
Document à titre d'information seulement. Le paiement officiel est indiqué sur l'autorisation de financement des IRSC.
