# Supplementary material for: The Good Food for Learning Universal Curriculum-Integrated Healthy School Lunch Intervention: Protocol for a Two-Year Matched Control Pre-Post and Case Study
Source: JMIR Res Protoc. 2021 Sep 21;10(9):e30899. doi: 10.2196/30899 (PMC8493466; doi:10.2196/30899)
Supplement: Multimedia Appendix 4 [file resprot_v10i9e30899_app4.pdf]

**Review Type / Type d'évaluation:**	Reviewer 1 / Évaluateur 1
**Name of Applicant / Nom du chercheur:**	Engler-Stringer, Rachel
**Application No. / Numéro de demande:**	425692
**Agency / Agence:**	CIHR/IRSC
**Competition / Concours:**	Project Grant/Subvention Projet
**Committee / Comité:**	Public, Community & Population Health/Santé publique, santé communautaire et santé des populations
**Title / Titre:**	Improving School Food: Universal Integrated Healthy Lunch Intervention Research

Adjudication Criteria/Critères de sélection

Significance and Impact of the Research/Importance et impact de la recherche: 4.2

Approaches and Methods/Approches et méthodes: 4.3

Expertise, Experience and Resources/Expertise, expérience et ressources: 4.4

Top/Bottom Selection/Groupe supérieur/inférieur

- (●) **Top/Groupe supérieur**
- ( ) **Bottom/Groupe inférieur**

**Review Type / Type d'évaluation:**	Reviewer 1 / Évaluateur 1
**Name of Applicant / Nom du chercheur:**	Engler-Stringer, Rachel
**Application No. / Numéro de demande:**	425692
**Agency / Agence:**	CIHR/IRSC
**Competition / Concours:**	Project Grant/Subvention Projet
**Committee / Comité:**	Public, Community & Population Health/Santé publique, santé communautaire et santé des populations
**Title / Titre:**	Improving School Food: Universal Integrated Healthy Lunch Intervention Research

Summary of Application/Résumé de la demande:

The purpose of this population health intervention research (PHIR) of a universal school based lunch program in two school in Saskatoon is to conduct case study of the process of implementation, benefits, challenges and perceptions of this program and to evaluate it through a non-blinded, experimental study with control group including a) plate-waste of lunches to examine contribution to overall diet quality and surveys of food-related knowledge, attitudes and practices. The proposal has four research questions:

RQ1: What are the benefits of and challenges faced in establishing and administering a universal curriculum-integrated healthy school lunch program?

RQ2: What is the difference in diet quality, food group, and key nutrient consumption among children in intervention schools after the implementation of a comprehensive, curriculum-integrated universal school lunch program, as compared to children in control schools?

RQ3: What is the difference in the food-related knowledge, attitudes, and practices among child participants in intervention schools after the implementation of a comprehensive, curriculum-integrated universal school lunch program, as compared to children in control schools?

RQ4: What aspects of curriculum integration show promise in enhancing food-related learning?

RQ1 and 4 will be accomplished via qualitative semi-structured interview and key-informant interviews and coded through qualitative analytic techniques (i.e., Qualitative data will be analyzed initially in an open coding process, to allow for emerging themes, followed by analysis using a priori generated code lists derived from the literature and our RQs.).

For RQ 2 & 3 two intervention schools and two control schools will be included. The intervention will be universal lunch program offered for two years to all student, while the control schools will continue with pre-existing small lunch program. Based on sample size calculations a minimum of 148 children from the intervention schools will be matched to 148 children in control schools. Pre-intervention and at the end of the intervention student's lunch consumption will be assessed via digital photography and plate-waste measurement assessment baseline and endpoint in both intervention and control schools we will administer a survey of food-related knowledge, attitudes and practices adapted from the Individual Eating Assessment Tool (IEAT) to all participating students in grades 4-6 in the intervention and control schools. Outcomes will be assessed via multi-level regression analysis accounting for student characteristics, interaction effects (i.e., gender) will also be explored.

**Review Type / Type d'évaluation:**	Reviewer 1 / Évaluateur 1
**Name of Applicant / Nom du chercheur:**	Engler-Stringer, Rachel
**Application No. / Numéro de demande:**	425692
**Agency / Agence:**	CIHR/IRSC
**Competition / Concours:**	Project Grant/Subvention Projet
**Committee / Comité:**	Public, Community & Population Health/Santé publique, santé communautaire et santé des populations
**Title / Titre:**	Improving School Food: Universal Integrated Healthy Lunch Intervention Research

Strengths and Weaknesses/Forces et faiblesses:

This is a well written multi-prong study that seek to evaluate a real world intervention assessing whether a universal based lunch program at two Saskatoon schools will improve child nutrition compared to control schools where there is only a small lunch program. The academic multi-disciplinary research team brings together population health, nutrition, nursing, education and sustainable food systems researchers. And there is excellent support from community, school district and other groups. While the intervention is complex and there are multiple RQ and measurement end points the intervention and research appears feasible given the intuitional support and additional funding. The applicants have carefully considered previous reviewer's comments.

Areas from improvement/clarity are relatively minor.

There remains some concerns around measurement bias as the students are aware that they are being studied. While the applicants state that they conduct the digital photography and plate waste analysis in a way that minimized the effect on student behaviour, it is it not clear that behaviour might not be more affected in the context of the intervention setting compared to the controls.

Expected participation rates seems a bit optimistic, but the they appears to be sufficient 'surplus' sample size to meet the size presented for sufficient power in the study. They also may not have perfect matches given the need to matched on median neighbourhood income, but would not present a major problem to the study design if not all student had a perfect match. It is also not clear how students leaving/moving will be dealt with? Does that effect sample size calculation? With these children still be followed, censored? The multi-level analysis plan seems fairly complex given the sample size . (e.g, children nested with classes within 4 schools) and RQ and do not necessarily strengthen the application

**Review Type / Type d'évaluation:**	Reviewer 1 / Évaluateur 1
**Name of Applicant / Nom du chercheur:**	Engler-Stringer, Rachel
**Application No. / Numéro de demande:**	425692
**Agency / Agence:**	CIHR/IRSC
**Competition / Concours:**	Project Grant/Subvention Projet
**Committee / Comité:**	Public, Community & Population Health/Santé publique, santé communautaire et santé des populations
**Title / Titre:**	Improving School Food: Universal Integrated Healthy Lunch Intervention Research

Budget Recommendation/Recommandation budgétaire:

None noted.

Please indicate your appraisal of the integration of sex as a biological variable as a strength, weakness, or not applicable to the proposal./Prière de sélectionner une option pour donner votre évaluation de l'intégration du sexe comme variable biologique en tant que point fort ou point faible de la proposition, ou en tant qu'élément non applicable à la proposition.

- ○ **Strength/Point fort**
- ○ **Weakness/Point faible**
- ◉ **Not applicable/Non applicable**

Please indicate your appraisal of the integration of gender as a socio-cultural determinant of health as a strength, weakness, or not applicable to the proposal./Prière de sélectionner une option pour donner votre évaluation de l'intégration du genre comme déterminant socioculturel de la santé en tant que point fort ou point faible de la proposition, ou en tant qu'élément non applicable à la proposition.

- ◉ **Strength/Point fort**
- ○ **Weakness/Point faible**
- ○ **Not applicable/Non applicable**

**Review Type / Type d'évaluation:**	Reviewer 1 / Évaluateur 1
**Name of Applicant / Nom du chercheur:**	Engler-Stringer, Rachel
**Application No. / Numéro de demande:**	425692
**Agency / Agence:**	CIHR/IRSC
**Competition / Concours:**	Project Grant/Subvention Projet
**Committee / Comité:**	Public, Community & Population Health/Santé publique, santé communautaire et santé des populations
**Title / Titre:**	Improving School Food: Universal Integrated Healthy Lunch Intervention Research

Sex and/or Gender Considerations/Notions de sexe et/ou de genre:

There is a relevant consideration of gender differences between boy and girls. The study is not designed or sufficiently powered to look a differences between sex assigned at birth and self identified gender.

**Review Type / Type d'évaluation:**	Reviewer 2 / Évaluateur 2
**Name of Applicant / Nom du chercheur:**	Engler-Stringer, Rachel
**Application No. / Numéro de demande:**	425692
**Agency / Agence:**	CIHR/IRSC
**Competition / Concours:**	Project Grant/Subvention Projet
**Committee / Comité:**	Public, Community & Population Health/Santé publique, santé communautaire et santé des populations
**Title / Titre:**	Improving School Food: Universal Integrated Healthy Lunch Intervention Research

Adjudication Criteria/Critères de sélection

Significance and Impact of the Research/Importance et impact de la recherche: 4.7

Approaches and Methods/Approches et méthodes: 4.7

Expertise, Experience and Resources/Expertise, expérience et ressources: 4.7

Top/Bottom Selection/Groupe supérieur/inférieur

- ◉ **Top/Groupe supérieur**
- ○ **Bottom/Groupe inférieur**

**Review Type / Type d'évaluation:**	Reviewer 2 / Évaluateur 2
**Name of Applicant / Nom du chercheur:**	Engler-Stringer, Rachel
**Application No. / Numéro de demande:**	425692
**Agency / Agence:**	CIHR/IRSC
**Competition / Concours:**	Project Grant/Subvention Projet
**Committee / Comité:**	Public, Community & Population Health/Santé publique, santé communautaire et santé des populations
**Title / Titre:**	Improving School Food: Universal Integrated Healthy Lunch Intervention Research

Summary of Application/Résumé de la demande:

This research proposal is proposing to test a universal integrated healthy lunch intervention

Strengths and Weaknesses/Forces et faiblesses:

Strengths: The biggest strength, although many could be named is the fact that this project is proposing a UNIVERSAL program.

The application is really well written making striking statements in the background section.

The authors addressed well the comments from previous rounds.

Already have funding for the idea

The extensive involvement with knowledge users.

The sample size is manageable

The ethics is already in place

Qualitative data will also be captured

**Review Type / Type d'évaluation:**	Reviewer 2 / Évaluateur 2
**Name of Applicant / Nom du chercheur:**	Engler-Stringer, Rachel
**Application No. / Numéro de demande:**	425692
**Agency / Agence:**	CIHR/IRSC
**Competition / Concours:**	Project Grant/Subvention Projet
**Committee / Comité:**	Public, Community & Population Health/Santé publique, santé communautaire et santé des populations
**Title / Titre:**	Improving School Food: Universal Integrated Healthy Lunch Intervention Research

Budget Recommendation/Recommandation budgétaire:

No issue with the budget

Please indicate your appraisal of the integration of sex as a biological variable as a strength, weakness, or not applicable to the proposal./Prière de sélectionner une option pour donner votre évaluation de l'intégration du sexe comme variable biologique en tant que point fort ou point faible de la proposition, ou en tant qu'élément non applicable à la proposition.

- (●) **Strength/Point fort**
- ( ) **Weakness/Point faible**
- ( ) **Not applicable/Non applicable**

Please indicate your appraisal of the integration of gender as a socio-cultural determinant of health as a strength, weakness, or not applicable to the proposal./Prière de sélectionner une option pour donner votre évaluation de l'intégration du genre comme déterminant socioculturel de la santé en tant que point fort ou point faible de la proposition, ou en tant qu'élément non applicable à la proposition.

- (●) **Strength/Point fort**
- ( ) **Weakness/Point faible**
- ( ) **Not applicable/Non applicable**

**Review Type / Type d'évaluation:**	Reviewer 2 / Évaluateur 2
**Name of Applicant / Nom du chercheur:**	Engler-Stringer, Rachel
**Application No. / Numéro de demande:**	425692
**Agency / Agence:**	CIHR/IRSC
**Competition / Concours:**	Project Grant/Subvention Projet
**Committee / Comité:**	Public, Community & Population Health/Santé publique, santé communautaire et santé des populations
**Title / Titre:**	Improving School Food: Universal Integrated Healthy Lunch Intervention Research

Sex and/or Gender Considerations/Notions de sexe et/ou de genre:

Well addressed

**Review Type / Type d'évaluation:**	Reviewer 3 / Évaluateur 3
**Name of Applicant / Nom du chercheur:**	Engler-Stringer, Rachel
**Application No. / Numéro de demande:**	425692
**Agency / Agence:**	CIHR/IRSC
**Competition / Concours:**	Project Grant/Subvention Projet
**Committee / Comité:**	Public, Community & Population Health/Santé publique, santé communautaire et santé des populations
**Title / Titre:**	Improving School Food: Universal Integrated Healthy Lunch Intervention Research

Adjudication Criteria/Critères de sélection

Significance and Impact of the Research/Importance et impact de la recherche: 4.0

Approaches and Methods/Approches et méthodes: 4.1

Expertise, Experience and Resources/Expertise, expérience et ressources: 4.5

Top/Bottom Selection/Groupe supérieur/inférieur

- (●) **Top/Groupe supérieur**
- ( ) **Bottom/Groupe inférieur**

**Review Type / Type d'évaluation:**	Reviewer 3 / Évaluateur 3
**Name of Applicant / Nom du chercheur:**	Engler-Stringer, Rachel
**Application No. / Numéro de demande:**	425692
**Agency / Agence:**	CIHR/IRSC
**Competition / Concours:**	Project Grant/Subvention Projet
**Committee / Comité:**	Public, Community & Population Health/Santé publique, santé communautaire et santé des populations
**Title / Titre:**	Improving School Food: Universal Integrated Healthy Lunch Intervention Research

Summary of Application/Résumé de la demande:

The purpose of this population health intervention research (a iKT project, with knowledge user-partner) is to study the impacts of a universal,curriculum-integrated healthy school lunch program in elementary schools in Saskatoon over a two-year period on food consumption, dietary quality and food and nutrition-related knowledge, attitudes and practices.

Strengths and Weaknesses/Forces et faiblesses:

Strengths:

This project has a very high potential public health impact. The comments from previous reviews have generally been very well addressed, and the project now includes a theoretical basis for the population-level intervention. The various phases are well described, appear feasible, with more than adequate justification and supporting tools such as lessons plans provided, and the instruments and methods should meet the stated process and outcome information that is sought, most importantly, the practical challenges to implementation.

School lunch program and curriculum was developed with stakeholders and researchers appear to be very responsive to their partners.

Weaknesses:

If the aim is to scale up and implement a universal, integrated school lunch program across all schools, an assessment of the feasibility at the ‘school population’ level seems to be missing – e.g., how many schools have the required kitchen and other necessary infrastructure needed? What about schools’ and families’ openness to what could be perceived as an intrusive intervention? Would this not also be key information prior to considering a province wide or scaling up intervention? Minimally, some easily obtained information could have been presented. The project is presented as a case study, but its potential reach would allow for a greater appreciation of the potential impact.

For unexplained reasons, the applicants went from 4 to only 2 intervention schools. This will further limit the ability to assess school level factors, especially since we already know that these are unique with respect to infrastructure and openness. It is unclear to what extent the information gathered will be applicable or generalizable, given that these were very keen schools selected by the school board.

More explicit feasibility measures, beyond what will be obtained qualitatively, would be helpful to include, as some of these could be quantified, e.g. proportion with complete data, etc.

It is unclear why, in addition to the plate waste images, overall intake (calories, or quality) is not assessed, for example with repeat 24 hour diet recalls. (For example, increasing physical activity at school has known consequences of reducing physical activity outside school, so that the overall gain is nil). Also, with only 2 meals assessed per study, the measures proposed may be appropriate to estimate school level outcomes, but not individual change- but how meaningful is this with only 2 control schools and 2 intervention schools? Can random effects MLM really be used with only 2 intervention schools, as described? If only classroom is the clustering variable, this may not be appropriate as the intervention is at the school level. Again, as this is a case study, this is not a major concern. The qualitative approach described – which focuses on documenting challenges to implementation - will yield useful albeit incomplete information.

The applicants mention the use of DAGS to identify confounders, but these were not provided.

Is there a possibility that the anonymous donor could be say a food vendor, looking for a contract, and would this be an issue?

Finally, cost does not seem to be assessed (or even the feasibility of obtaining cost)- should this be considered?

**Review Type / Type d'évaluation:**	Reviewer 3 / Évaluateur 3
**Name of Applicant / Nom du chercheur:**	Engler-Stringer, Rachel
**Application No. / Numéro de demande:**	425692
**Agency / Agence:**	CIHR/IRSC
**Competition / Concours:**	Project Grant/Subvention Projet
**Committee / Comité:**	Public, Community & Population Health/Santé publique, santé communautaire et santé des populations
**Title / Titre:**	Improving School Food: Universal Integrated Healthy Lunch Intervention Research

Budget Recommendation/Recommandation budgétaire:

Justified

Please indicate your appraisal of the integration of sex as a biological variable as a strength, weakness, or not applicable to the proposal./Prière de sélectionner une option pour donner votre évaluation de l'intégration du sexe comme variable biologique en tant que point fort ou point faible de la proposition, ou en tant qu'élément non applicable à la proposition.

- (●) **Strength/Point fort**
- ( ) **Weakness/Point faible**
- ( ) **Not applicable/Non applicable**

Please indicate your appraisal of the integration of gender as a socio-cultural determinant of health as a strength, weakness, or not applicable to the proposal./Prière de sélectionner une option pour donner votre évaluation de l'intégration du genre comme déterminant socioculturel de la santé en tant que point fort ou point faible de la proposition, ou en tant qu'élément non applicable à la proposition.

- (●) **Strength/Point fort**
- ( ) **Weakness/Point faible**
- ( ) **Not applicable/Non applicable**

**Review Type / Type d'évaluation:**	Reviewer 3 / Évaluateur 3
**Name of Applicant / Nom du chercheur:**	Engler-Stringer, Rachel
**Application No. / Numéro de demande:**	425692
**Agency / Agence:**	CIHR/IRSC
**Competition / Concours:**	Project Grant/Subvention Projet
**Committee / Comité:**	Public, Community & Population Health/Santé publique, santé communautaire et santé des populations
**Title / Titre:**	Improving School Food: Universal Integrated Healthy Lunch Intervention Research

Sex and/or Gender Considerations/Notions de sexe et/ou de genre:

Both are addressed in the proposal.
