# Supplementary material for: The Good Food for Learning Universal Curriculum-Integrated Healthy School Lunch Intervention: Protocol for a Two-Year Matched Control Pre-Post and Case Study
Source: JMIR Res Protoc. 2021 Sep 21;10(9):e30899. doi: 10.2196/30899 (PMC8493466; doi:10.2196/30899)
Supplement: Multimedia Appendix 2 [file resprot_v10i9e30899_app2.pdf]

Canadian Institutes of Health Research
160 Elgin Street, 9th Floor
Address Locator 4809A
Ottawa, Ontario K1A 0W9

Instituts de recherche en santé du Canada
160, rue Elgin, 9e étage
Indice de l'adresse 4809A
Ottawa (Ontario) K1A 0W9

Institute of Aging
Institute of Cancer Research
Institute of Circulatory and Respiratory Health
Institute of Gender and Health
Institute of Genetics
Institute of Health Services and Policy Research
Institute of Human Development and Child and Youth Health
Institute of Indigenous Peoples' Health
Institute of Infection and Immunity
Institute of Musculoskeletal Health and Arthritis
Institute of Neurosciences, Mental Health and Addiction
Institute of Nutrition, Metabolism and Diabetes
Institute of Population and Public Health

Institut du vieillissement
Institut du cancer
Institut de la santé circulatoire et respiratoire
Institut de la santé des femmes et des hommes
Institut de génétique
Institut des services et des politiques de la santé
Institut du développement et de la santé des enfants et des adolescents
Institut de la santé des Autochtones
Institut des maladies infectieuses et immunitaires
Institut de l'appareil locomoteur et de l'arthrite
Institut des neurosciences, de la santé mentale et des toxicomanies
Institut de la nutrition, du métabolisme et du diabète
Institut de la santé publique et des populations

January 22, 2020

Dr. Rachel Rosa Engler-Stringer
Community Health and Epidemiology
College of Medicine
University of Saskatchewan
104 Clinic Place
Saskatoon, Saskatchewan S7N 5E5

Dear Dr. Engler-Stringer,

On behalf of the Canadian Institutes of Health Research (CIHR), I am pleased to inform you that your application entitled "Improving School Food: Universal Integrated Healthy Lunch Intervention Research", submitted to the Project Grant: Fall 2019 competition, has been approved for funding.

Your application reviews and competition results are available through ResearchNet. If you are unable to view these documents, please contact us at support-soutien@cihr-irsc.gc.ca. Your Authorization for Funding will follow in the mail.

Of note, the number of approved applications (as indicated in the Notice of Decision) includes applications that fell above the funding cutoff for the committee and, if applicable, any Early Career Researcher (ECR) application(s) that fell below the funding cutoff but were fully funded through the funds allotted specifically for equalization of success rates of ECRs. Through this equalization process, CIHR ensures that funding of ECRs is at an equal proportion as ECR applicants within the Project Grant competition, or, in other words, that ECR applicants have similar success rate as mid-career or senior applicants.

As CIHR does not notify co-applicants of the decision, we ask that you inform those individuals involved, along with their research institutions (if different from your own) of the outcome of this application.

Should you have any questions, please do not hesitate to communicate with a Processing Officer in the Contact Centre at 613-954-1968 or by e-mail at support-soutien@cihr-irsc.gc.ca.

Congratulations on your success in this competition.

Sincerely,

C. Leneï

Chaidwick Leneïs
Manager, Program Design and Delivery
Research Programs Portfolio

491393-201909PJT-PH1-425692-117642-DLPJT

CIHR IRSC
Discoveries for life / Découvertes pour la vie

Canada
